# Research on the dynamic behavior of flexible drilling tools in ultrashort-radius radial horizontal wells

**DOI:** 10.1038/s41598-024-57742-3

**Published:** 2024-03-27

**Authors:** Zhiqiang Lin, Min Luo, Jing Wang, Tingting Xiu, Qiaozhen Li

**Affiliations:** 1 School of Mechanical Science and Engineering, Northeast Petroleum University, Daqing, China; 2 Mathematics and Statistics College, Northeast Petroleum University, Daqing, China

**Keywords:** Ultrashort radius radial horizontal well, Flexible drill tool, Flexible drill pipe, Guide pipe, Multilayer contact, Energy science and technology, Engineering

## Abstract

A flexible drilling tool is a special drilling tool for ultrashort-radius radial horizontal wells. This tool is composed of many parts and has the characteristics of a multibody system. In this paper, a numerical method for the dynamic analysis of flexible drilling tools is proposed. The flexible drill tool is discretized into spatial beam elements, while the multilayer contact of the flexible drilling tool is represented by the multilayer dynamic gap element, and the dynamic model of the multibody system for the flexible drilling tool’s multilayer contact is established, considering the interaction force between the drill bit and the rock. The nonlinear dynamic equation is solved using the Newmark method and Newton–Raphson method. An analysis of the dynamic behavior of a flexible drilling tool is conducted. The results indicate that the flexible drilling tool experiences vortex formation due to the interaction between the flexible drilling pipe and the guide pipe, leading to increased friction and wear. This situation hinders safe drilling operations with flexible drilling tools. The collision force of the flexible drilling tool near the bottom of the hole is more severe than that of the other tool types, which may lead to failure of the connection.

## Introduction

The technology of ultrashort-radius horizontal branch wells plays an active role in increasing the production of old wells and improving development^1^. The use of special flexible drilling tools is necessary to meet the requirement of a curvature radius less than 4 m. The concept of flexible drilling tools was first proposed in the 1990s^2,3^. To meet the requirements of a high-build slope, a flexible drill is designed for use in a multistage articulated structure. Liu designed a ball basket universal joint and cross-joint structure to realize rotation between the units, and several indoor experiments were performed to test the sealing performance under actual operating pressure and tensile strength of prototyping tools^4,5^. Wang conducted the bionic design and trial production of drill pipe joints based on the principles of engineering bionics^6^. The drill system developed by Arif Gok and Kadir Gok^7,8^ features a closed-circuit cooling system, which effectively reduces the undesired temperature rise during the drilling process.

**Figure 1:**
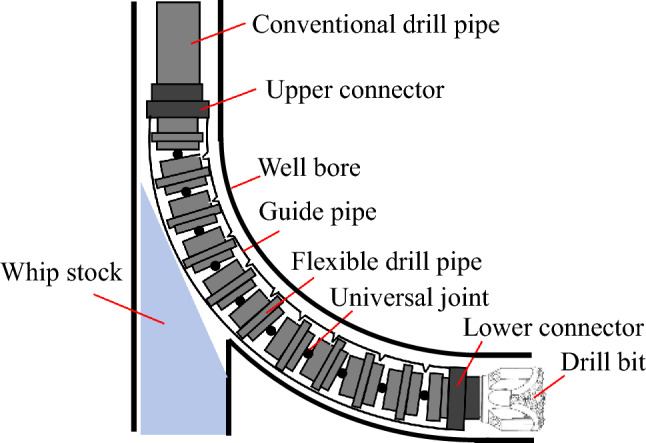
Flexible drill tool drilling.

The flexible drill tool is composed mainly of a flexible drill pipe and a guide pipe (Fig. 1). The flexible drill pipe is assembled inside the guide pipe, and the two ends are connected through the connector of the drill tool. During drilling, the weight on the bit is transferred to the guide pipe through the connector, while the torque is transferred to the flexible drill pipe. Due to the large number of flexible drill parts and complex structure, the strength of flexible drills has been a concern. The finite element method was employed by Zhu to conduct mechanical analysis of flexible joints in flexible drilling tools. It was shown that the ball key is the most dangerous part of the flexible joint, and its safety can be improved by increasing the diameter of the ball key^9^. Wang conducted a kinematic analysis of the flexible drill pipe as a whole and a static analysis of the local part^10^. The structural parameter design method of a flexible ball cage joint was established by Zhu based on geometric coordination and material strength theory. The multibody motion process was further analysed using the multibody dynamics method, studying motion characteristics such as the collision contact force, constant velocity characteristic, transfer efficiency, and deflection moment^11^. Luo and Xu utilized the finite element method to establish a numerical calculation approach for the contact nonlinear analysis of a controllable universal articulated flexible rod in a pipe. They introduced the dynamic relaxation method to solve the numerical model, which addressed the issue of nonconvergence in the nonlinear model caused by rigid body displacement in beam-beam contact problems. The accuracy of this model was verified through a numerical example^12–14^. In summary, the majority of existing studies have focused primarily on static strength analysis and motion analysis of local models for flexible drilling tools. Although some scholars have investigated the load transfer law of entire flexible drilling tools, their numerical approach employs high damping to suppress vibration and obtain static results. While this method effectively addresses convergence issues in obtaining solutions, it fails to accurately capture the dynamic characteristics of the hinged flexible rod. Moreover, this approach does not consider the drill's interaction with the rock. The dynamic characteristics of drilling tools significantly impact their working conditions and safety^15–17^.

The purpose of this article is to investigate the dynamic problem of multi-body flexible drilling system in borehole. Based on previous studies, we developed a numerical calculation method suitable for dynamic contact nonlinear analysis of a multibody system comprising flexible drilling tools.The flexible drill tool is discretized intospatial beam elements, while the multilayer contact of the flexible drilling tool is represented by the multilayer dynamic gap element, and the dynamic model of the multibody system for the flexible drilling tool's multilayer contact is established, considering the interaction force between the drill bit and the rock The nonlinear dynamic equation is solved using the Newmark method and Newton–Raphson method. This research can provide an effective numerical simulation method for pre-drilling simulation and prediction of flexible drilling tools.

## Model and method

### Assumptions

The flexible drill pipe is a hinged beam with a variable section.

(2) The guide pipe is a continuous beam with equal cross-sections.
(3) The flexible drill pipe and guide pipe are linearly elastic.
(4) The flexible drill pipe and guide pipe are assumed to have small deformations.
(5) The joint is rigid.
(6) The flexible drill pipe and guide pipe before deformation are located in the center of the borehole.

### Mechanical model

**Figure 2:**
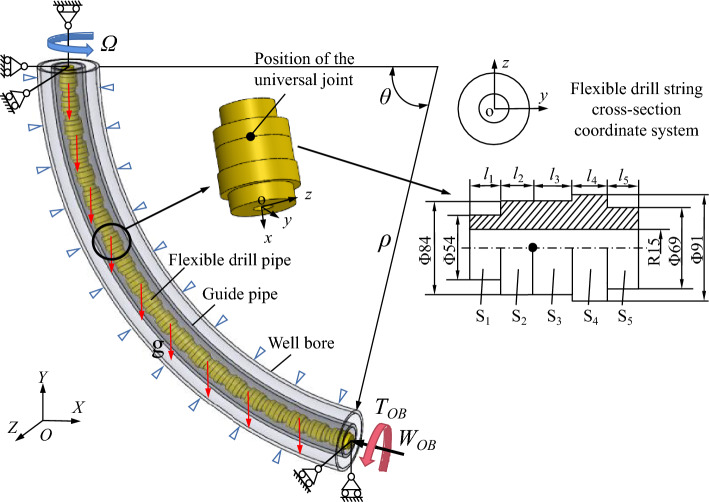
Flexible drilling tool dynamics model.

The dynamic model of flexible drilling tools is illustrated in Fig. 2. At the top, a constant rotational speed ${\Omega }$ is maintained on the flexible drill pipe. At the bottom, a bit-rock interaction model is added. The displacement boundary conditions for flexible drilling tools are presented in Table 1.

**Table 1:** The displacement boundary conditions of the flexible drilling tool.

Items	Constrained position	UX	UY	UZ	ROTX	ROTY	ROTZ
Flexible drill pipe	Top	√	√	√	–	–	–
	Bottom		√	√	–	–	–
Guide pipe	Top	√	√	√	–	–	–
	Bottom		√	√	–	–	–
Wellbore	All nodes	√	√	√	√	√	√

A simplified model of drill bit rock interaction is applied to the bottom of the flexible drill pipe^18^. The calculation formula is shown in Eq. (1):

1 $$ T_{OB} = \frac{{\eta D_{{{\text{bit}}}} W_{OB} }}{3} $$

where $T_{OB}$ is the total torque generated by the friction of the drill bit, $\eta$ is the dynamic friction factor between the bit and the rock, $D_{{{\text{bit}}}}$ is the bit diameter, and $W_{OB}$ is the weight of the bit.

### Dynamic finite element model

**Figure 3:**
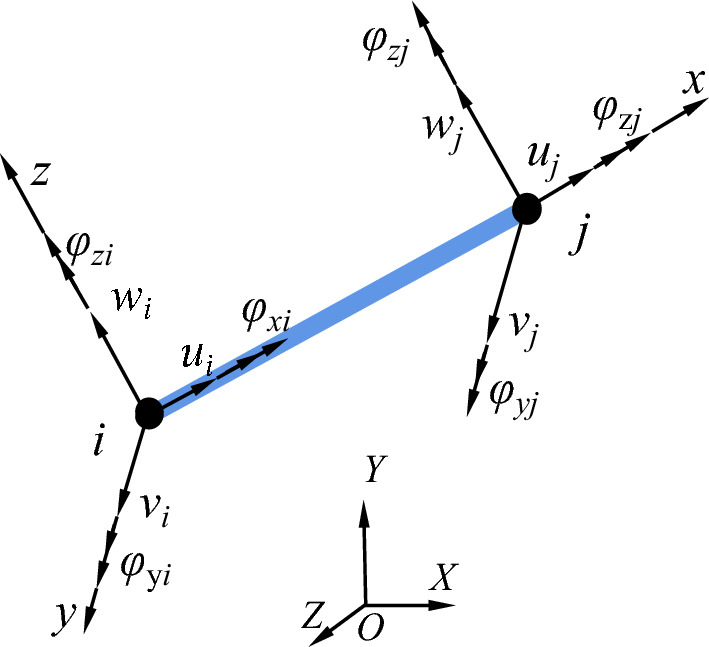
Spatial beam element.

The flexible drilling tool is a complex structure that can be effectively analyzed using the finite element method^19^. The flexible drilling tool is a slender structure, and a beam element is used for dispersion. Figure 3 shows a spatial beam element. The spatial beam element has 12 nodal coordinates that describe the translations and slopes of the two nodes^20^.

The displacement vector of the spatial beam element nodes is as follows:

2 $$ {\mathbf{q}}^{e} = [u_{{1}} ,v_{{1}} ,w_{{1}} ,\varphi_{{x{1}}} ,\varphi_{{y{1}}} ,\varphi_{{z{1}}} ,u_{{2}} ,v_{{2}} ,w_{{2}} ,\varphi_{{x{2}}} ,\varphi_{{y{2}}} ,\varphi_{{z{2}}} ]^{{\text{T}}} $$

The displacement of any point in the spatial beam element is:

3 $$ {\mathbf{d}}^{{\text{e}}} { = }{\mathbf{Nq}}^{{\text{e}}} $$

where ${\mathbf{N}}$ is the shape function matrix.

4 $$ {\mathbf{N}}^{{\text{T}}} { = }\left[ {\begin{array}{*{20}c} {1 - \xi } & 0 & 0 & 0 \\ 0 & {1 - 3\xi^{2} + 2\xi^{3} } & 0 & 0 \\ 0 & 0 & {1 - 3\xi^{2} + 2\xi^{3} } & 0 \\ 0 & 0 & 0 & {1 - \xi } \\ 0 & 0 & {(1 + 2\xi - \xi^{2} )\xi l} & 0 \\ 0 & {(1 - 2\xi + \xi^{2} )\xi l} & 0 & 0 \\ \xi & 0 & 0 & 0 \\ 0 & {3\xi^{2} - 2\xi^{3} } & 0 & 0 \\ 0 & 0 & {3\xi^{2} - 2\xi^{3} } & 0 \\ 0 & 0 & 0 & \xi \\ 0 & 0 & {{(}\xi^{2} - \xi^{3} {)}l} & 0 \\ 0 & {{(} - \xi^{2} + \xi^{3} {)}l} & 0 & 0 \\ \end{array} } \right] $$

where $$\xi { = }\frac{x}{l}$$.

The stiffness matrix and mass matrix of the space beam element are

5 $$ {\mathbf{M}}_{{}}^{e} = \int_{{0}}^{l} {\rho A{\mathbf{N}}_{{}}^{{\text{T}}} {\mathbf{N}}{\text{d}}x} $$

6 $$ {\mathbf{K}}_{{}}^{e} = \int_{{0}}^{l} {{(}{\mathbf{HN}}{)}^{{\text{T}}} {\mathbf{DHN}}{\text{d}}x} $$

where ${\mathbf{H}}$ is the matrix of differential operators and ${\mathbf{D}}$ is the elastic matrix.

$$ {\mathbf{H}}{ = }\left[ {\begin{array}{*{20}c} {\frac{{\text{d}}}{{{\text{d}}x}}} & {0} & {0} & {0} \\ {0} & {\frac{{\text{d}}}{{{\text{d}}x^{{2}} }}} & {0} & {0} \\ {0} & {0} & {\frac{{\text{d}}}{{{\text{d}}x^{{2}} }}} & {0} \\ {0} & {0} & {0} & {\frac{{\text{d}}}{{{\text{d}}x}}} \\ \end{array} } \right],{\mathbf{D}} = \left[ {\begin{array}{*{20}c} {EA} & {0} & {0} & {0} \\ {0} & {EI_{z} } & {0} & {0} \\ {0} & {0} & {EI_{y} } & {0} \\ {0} & {0} & {0} & {GI_{x} } \\ \end{array} } \right] $$

where $A$ is the section area of the space beam element; $I_{z}$ and $I_{y}$ are the second moments of the area about the neutral axes *z* and *y*, respectively; and $I_{x}$ is the polar moment of inertia of the area.

The damping matrix is damped proportionally.

7 $$ {\mathbf{C}}_{{}}^{e} = \alpha {\mathbf{M}}_{{}}^{e} + \beta {\mathbf{K}}_{{}}^{e} $$

The relationship between the components of a spatial beam element and the components of the global coordinate system is described in terms of the local coordinate system.

8 $$ {\overline{\mathbf{q}}}^{e} { = }{\mathbf{\Gamma q}}^{e} $$

9 $$ {\overline{\mathbf{K}}}_{{}}^{e} { = }{{\varvec{\Gamma}}}^{{\text{T}}} {\mathbf{K}}^{e} {{\varvec{\Gamma}}} $$

where the coordinate transformation matrix ${{\varvec{\Gamma}}}$.

$${{\varvec{\Gamma}}}{ = }\left[ {\begin{array}{*{20}c} {{\varvec{\upzeta}}} & 0 & 0 & 0 \\ 0 & {{\varvec{\upzeta}}} & 0 & 0 \\ 0 & 0 & {{\varvec{\upzeta}}} & 0 \\ 0 & 0 & 0 & {{\varvec{\upzeta}}} \\ \end{array} } \right],{{\varvec{\upzeta}}}{ = }\left[ {\begin{array}{*{20}c} {{\text{cos(}}X{,}x{)}} & {{\text{cos(}}X{,}y{)}} & {{\text{cos(}}X{,}z{)}} \\ {{\text{cos(}}Y{,}x{)}} & {{\text{cos(}}Y{,}y{)}} & {{\text{cos(}}Y{,}z{)}} \\ {{\text{cos(}}Z{,}x{)}} & {{\text{cos(}}Z{,}y{)}} & {{\text{cos(}}Z{,}z{)}} \\ \end{array} } \right]$$

where ${(}X{,}x{)}$ represents the angle between the global coordinate axis and the local coordinate axis.

**Figure 4:**
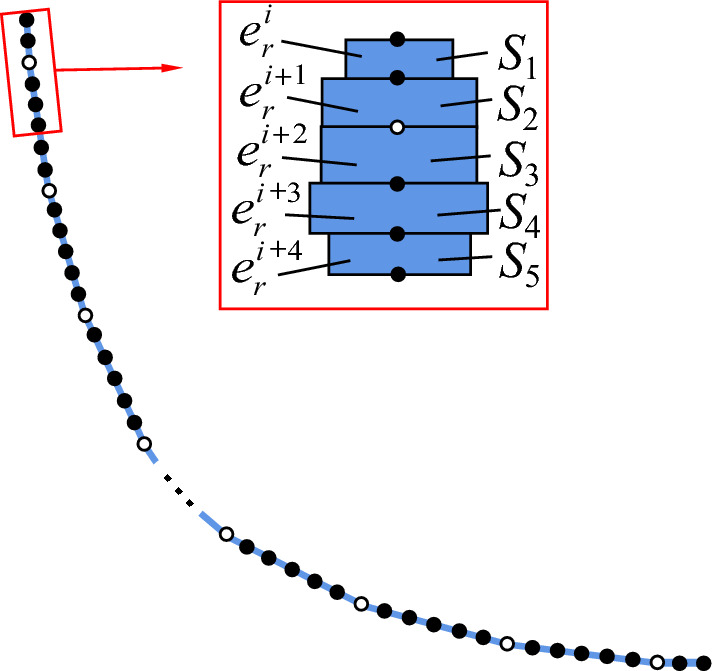
Finite element model of flexible drill pipe.

The finite element model of the flexible drill pipe, as depicted in Fig. 4, consists of five discrete space beam elements representing each section of the pipe based on its cross-section. The hinge point is located between elements $e_{{i + {1}}}^{r}$ and $e_{i + 2}^{r}$. Similarly, the guide pipe is discretized into equal sections using beam elements with an equivalent number of nodes as the flexible drill pipe.

The stiffness matrix, mass matrix and damping matrix of the flexible drill pipe and guide pipe are assembled according to the calculation formula of the spatial beam element.

$$ {\mathbf{K}}_{r} = \sum\limits_{e} {{\mathbf{K}}_{r}^{e} } ,{\mathbf{M}}_{r} = \sum\limits_{e} {{\mathbf{M}}_{r}^{e} } ,{\mathbf{C}}_{r} = \sum\limits_{e} {{\mathbf{C}}_{r}^{e} } $$

$$ {\mathbf{K}}_{d} = \sum\limits_{e} {{\mathbf{K}}_{d}^{e} } ,{\mathbf{M}}_{d} = \sum\limits_{e} {{\mathbf{M}}_{d}^{e} } ,{\mathbf{C}}_{d} = \sum\limits_{e} {{\mathbf{C}}_{d}^{e} } $$

where ${\mathbf{K}}_{r}$, ${\mathbf{M}}_{r}$ and ${\mathbf{C}}_{r}$ are the global stiffness matrix, the mass matrix and the damping matrix of the flexible drill pipe, respectively. ${\mathbf{K}}_{d}$, ${\mathbf{M}}_{d}$ and ${\mathbf{C}}_{d}$ are the global stiffness matrix, the mass matrix and the damping matrix of the guide pipe, respectively.

By disregarding the flexible pipe, interpipe contact, and interaction between the pipe run and borehole wall, we can independently formulate the dynamic equation of the flexible tool.

10 $$ \left\{ {\left[ {\begin{array}{*{20}c} {{\mathbf{M}}_{r} } & 0 \\ 0 & {{\mathbf{M}}_{d} } \\ \end{array} } \right]\left\{ \begin{gathered} {\ddot{\mathbf{q}}}_{r} (t) \hfill \\ {\ddot{\mathbf{q}}}_{d} (t) \hfill \\ \end{gathered} \right\} + \left[ {\begin{array}{*{20}c} {{\mathbf{C}}_{r} } & 0 \\ 0 & {{\mathbf{C}}_{d} } \\ \end{array} } \right]\left\{ \begin{gathered} {\dot{\mathbf{q}}}_{r} (t) \hfill \\ {\dot{\mathbf{q}}}_{d} (t) \hfill \\ \end{gathered} \right\} + \left[ {\begin{array}{*{20}c} {{\mathbf{K}}_{r} } & 0 \\ 0 & {{\mathbf{K}}_{d} } \\ \end{array} } \right]\left\{ \begin{gathered} {\mathbf{q}}_{r} (t) \hfill \\ {\mathbf{q}}_{d} (t) \hfill \\ \end{gathered} \right\} = \left[ \begin{gathered} {\mathbf{F}}_{r} \hfill \\ {\mathbf{F}}_{d} \hfill \\ \end{gathered} \right]} \right. $$

where ${\ddot{\mathbf{q}}}_{r} (t)$, ${\dot{\mathbf{q}}}_{r} (t)$ and ${\mathbf{q}}_{r} (t)$ are the acceleration array, the velocity array and the displacement array of the flexible drill pipe, respectively. ${\ddot{\mathbf{q}}}_{{\text{d}}} (t)$, ${\dot{\mathbf{q}}}_{{\text{d}}} (t)$ and ${\mathbf{q}}_{{\text{d}}} (t)$ are the acceleration array, the velocity array and the displacement array of the guide pipe, respectively. ${\mathbf{F}}_{r}$ and ${\mathbf{F}}_{d}$ are the equivalent nodal force arrays of the flexible drill pipe and guide pipe, respectively.

**Figure 5:**
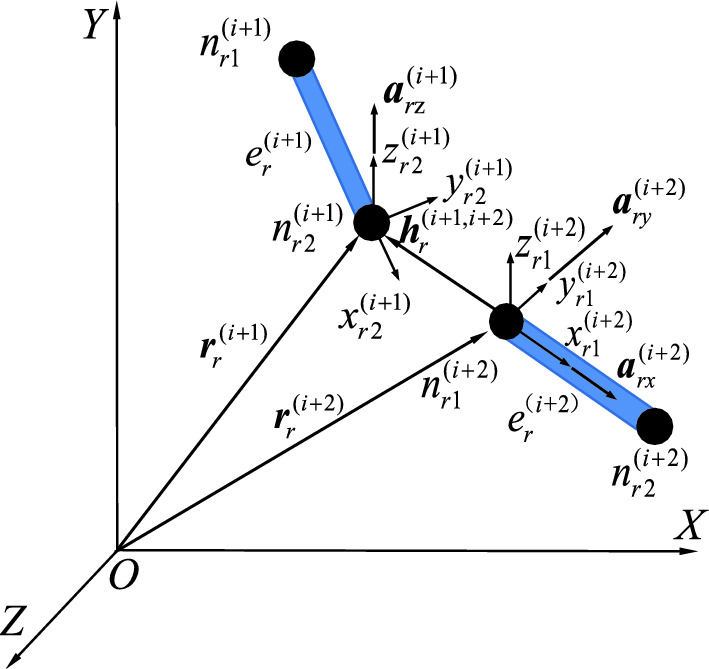
Vector diagram of controllable hinge points.

The kinematic constraints imposed by joint points have not been taken into account in the previously mentioned dynamic equations of flexible drill pipes. The coordinate systems $n_{r2}^{(i + 1)} x_{r2}^{(i + 1)} y_{r2}^{(i + 1)} z_{r2}^{(i + 1)}$ and $n_{r1}^{(i + 2)} x_{r1}^{(i + 2)} y_{r1}^{(i + 2)} z_{r1}^{(i + 2)}$ for the joint points of the flexible drill pipe should be established at nodes $n_{r2}^{(i + 1)}$ and $n_{r1}^{r(i + 2)}$. The linkage between beam elements $e_{r}^{{{(}i + 1{)}}}$ and $e_{r}^{{{(}i + 2{)}}}$ is established through the imposition of motion constraints on nodes $n_{r2}^{(i + 1)}$ and $n_{r1}^{(i + 2)}$. The element vectors ${\mathbf{a}}_{rz}^{{{(}i + 1{)}}}$, ${\mathbf{a}}_{ry}^{{{(}i + 2{)}}}$, and ${\mathbf{a}}_{rx}^{{{(}i + 2{)}}}$ are consolidated on element $e_{r}^{{{(}i + 1{)}}}$ (node $n_{r2}^{(i + 1)}$) and unit $e_{r}^{{{(}i + 2{)}}}$ (node $n_{r1}^{(i + 2)}$), and the vector ${\mathbf{h}}_{r}^{(i + 1,i + 2)}$ connects the joint points. The kinematic constraint equation of articulation can be defined by the kinematic relation between the vectors shown in Fig. 5.

The radius vectors of nodes $n_{r2}^{(i + 1)}$ and $n_{r1}^{(i + 2)}$ with respect to the origin O in the global coordinate system are determined as follows:

11 $$ \left\{ \begin{gathered} {\mathbf{r}}_{{\text{r}}}^{(i + 1)} = {\mathbf{\Lambda N\overline{q}}}_{{\text{r}}}^{(i + 1)} \hfill \\ {\mathbf{r}}_{{\text{r}}}^{(i + 2)} = {\mathbf{\Lambda N\overline{q}}}_{{\text{r}}}^{(i + 2)} \hfill \\ \end{gathered} \right. $$

The vector diameters of the hinge point coordinate systems $n_{r2}^{(i + 1)} x_{r2}^{(i + 1)} y_{r2}^{(i + 1)} z_{r2}^{(i + 1)}$ and $n_{r1}^{(i + 2)} x_{r1}^{(i + 2)} y_{r1}^{(i + 2)} z_{r1}^{(i + 2)}$, with respect to the origin O of the global coordinate system, are denoted by ${\mathbf{r}}_{{\text{r}}}^{(i + 1)}$ and ${\mathbf{r}}_{{\text{r}}}^{(i + 2)}$, respectively. ${{\varvec{\Lambda}}}$ is the cosine matrix of the deformed hinge coordinate system relative to the element coordinate system. ${\overline{\mathbf{q}}}_{{\text{r}}}^{(i + 1)}$ and ${\overline{\mathbf{q}}}_{{\text{r}}}^{{{ - }i + 2)}}$ represent the displacement vectors of nodes $n_{r2}^{(i + 1)}$ and $n_{r1}^{(i + 2)}$, respectively. The node-relative translational constraints at the locations where elements $e_{r}^{{{(}i + 1{)}}}$ and $e_{r}^{{{(}i + 2{)}}}$ coincide can be determined by constraining both the magnitude and direction of the vector ${\mathbf{h}}_{r}^{(i + 1,i + 2)} { = }\overrightarrow {{n_{r2}^{(i + 1)} n_{r1}^{(i + 2)} }}$.

12 $$ {\mathbf{h}}_{r}^{(i + 1,i + 2)} = {\mathbf{r}}_{r}^{(i + 1)} - {\mathbf{r}}_{r}^{(i + 2)} = 0 $$

The rotational constraints for elements $e_{{i + {1}}}^{r}$ and $e_{i + 2}^{r}$ are as follows:

13 $$ {{\varvec{\Phi}}}_{r} = \left[ {\begin{array}{*{20}c} {{\mathbf{h}}_{r}^{(i + 1)} } \\ {{\mathbf{a}}_{{r{\text{z}}}}^{{{(}i + 1{\text{)T}}}} {\mathbf{a}}_{ry}^{{{(}i + 2{)}}} } \\ {{\mathbf{a}}_{{r{\text{z}}}}^{{{(}i + 1{\text{)T}}}} {\mathbf{a}}_{rx}^{{{(}i + 2{)}}} - \cos \theta } \\ \end{array} } \right] = \left[ {\begin{array}{*{20}c} 0 \\ 0 \\ 0 \\ \end{array} } \right] $$

If the flexible drill pipe is connected by k hinges, then the overall articulated constraint equation of the flexible drill pipe can be derived.

14 $$ {{\varvec{\Phi}}}_{r} ({\mathbf{q}}_{r} ,t) = \left[ {\begin{array}{*{20}c} {{{\varvec{\Phi}}}_{r}^{1} ({\mathbf{q}}_{r}^{{}} ,t)} \\ {{{\varvec{\Phi}}}_{r}^{2} ({\mathbf{q}}_{r}^{{}} ,t)} \\ \vdots \\ {{{\varvec{\Phi}}}_{r}^{k} ({\mathbf{q}}_{r}^{{}} ,t)} \\ \end{array} } \right] = 0 $$

Equation (14) takes the derivative of time, and the velocity constraint equation is

15 $$ {{\varvec{\Phi}}}_{{r{\mathbf{q}}}}^{{}} {\dot{\mathbf{q}}}_{r} + {{\varvec{\Phi}}}_{t}^{{}} = 0 $$

where

16 $$ {{\varvec{\Phi}}}_{{r{\mathbf{q}}}}^{{}} { = }\left[ {\begin{array}{*{20}c} {\frac{{\partial \varvec{\Phi }_{r1} }}{{\partial q_{1} }}} & {\frac{{\partial \varvec{\Phi }_{r1} }}{{\partial q_{2} }}} & \cdots & {\frac{{\partial \varvec{\Phi }_{r1} }}{{\partial q_{n} }}} \\ {\frac{{\partial \varvec{\Phi }_{r2} }}{{\partial q_{1} }}} & {\frac{{\partial \varvec{\Phi }_{r2} }}{{\partial q_{2} }}} & \cdots & {\frac{{\partial \varvec{\Phi }_{r2} }}{{\partial q_{n} }}} \\ \vdots & \vdots & {} & \vdots \\ {\frac{{\partial \varvec{\Phi }_{rk} }}{{\partial q_{1} }}} & {\frac{{\partial \varvec1{\Phi }_{rk} }}{{\partial q_{2} }}} & \cdots & {\frac{{\partial \varvec{\Phi }_{rk} }}{{\partial q_{n} }}} \\ \end{array} } \right],{{\varvec{\Phi}}}_{t}^{{}} { = }\left[ {\begin{array}{*{20}c} {\frac{{\partial \varvec{\Phi }_{r1} }}{\partial t}} \\ {\frac{{\partial \varvec{\Phi }_{r2} }}{\partial t}} \\ \vdots \\ {\frac{{\partial \varvec{\Phi }_{rk} }}{\partial t}} \\ \end{array} } \right] $$

The Lagrange multiplier method is employed to incorporate the constraint equation into the overall motion equation of the flexible drill pipe, which can be derived as follows:

17 $$ \left\{ {\begin{array}{*{20}c} {\left[ {\begin{array}{*{20}c} {{\mathbf{M}}_{r} } & 0 \\ 0 & {{\mathbf{M}}_{d} } \\ \end{array} } \right]\left\{ \begin{gathered} {\ddot{\mathbf{q}}}_{r} (t) \hfill \\ {\ddot{\mathbf{q}}}_{d} (t) \hfill \\ \end{gathered} \right\} + \left[ {\begin{array}{*{20}c} {{\mathbf{C}}_{r} } & 0 \\ 0 & {{\mathbf{C}}_{d} } \\ \end{array} } \right]\left\{ \begin{gathered} {\dot{\mathbf{q}}}_{r} (t) \hfill \\ {\dot{\mathbf{q}}}_{d} (t) \hfill \\ \end{gathered} \right\} + \left[ {\begin{array}{*{20}c} {{\mathbf{K}}_{r} } & 0 \\ 0 & {{\mathbf{K}}_{d} } \\ \end{array} } \right]\left\{ \begin{gathered} {\mathbf{q}}_{r} (t) \hfill \\ {\mathbf{q}}_{d} (t) \hfill \\ \end{gathered} \right\} + \left[ {\begin{array}{*{20}c} {{{\varvec{\Phi}}}_{{{\text{r}}{\mathbf{q}}}}^{{\mathbf{T}}} } & 0 \\ 0 & 0 \\ \end{array} } \right]\left\{ \begin{gathered} {{\varvec{\uplambda}}}_{r}^{joint} \hfill \\ 0 \hfill \\ \end{gathered} \right\} = \left[ \begin{gathered} {\mathbf{F}}_{r} \hfill \\ {\mathbf{F}}_{d} \hfill \\ \end{gathered} \right]} \\ {{{\varvec{\Phi}}}_{r} ({\mathbf{q}},t) = 0} \\ \end{array} } \right. $$

The symbol ${{\varvec{\Phi}}}_{r} ({\mathbf{q}}_{{\text{r}}} ,t)$ denotes the set of constraint equations governing the flexible pipe within the universal joint, while ${{\varvec{\Phi}}}_{{{\text{r}}{\mathbf{q}}}}^{{\mathbf{T}}}$ represents the constraint equations associated with the Jacobian matrix. Additionally, ${{\varvec{\uplambda}}}_{r}^{joint}$ signifies the Lagrange multiplier array pertaining to hinged constraints.

The Lagrange multiplier matrix associated with it is derived:

18 $$ {{\varvec{\uplambda}}}_{r}^{joint} { = }\left[ {\begin{array}{*{20}c} {\varvec{\lambda }_{1} } & {\varvec{\lambda }_{2} } & \cdots & {\varvec{\lambda }_{k} } \\ \end{array} } \right]^{{\text{T}}} $$

Equation (17) is abbreviated as follows:

19 $$ \left\{ {\begin{array}{*{20}c} {{\mathbf{M}}_{{{\text{rd}}}} {\ddot{\mathbf{q}}}_{{{\text{rd}}}} (t) + {\mathbf{C}}_{{{\text{rd}}}} {\dot{\mathbf{q}}}_{{{\text{rd}}}} (t) + {\mathbf{K}}_{{{\text{rd}}}} {\mathbf{q}}_{{{\text{rd}}}} (t) + {{\varvec{\Phi}}}_{{{\text{rd}}{\mathbf{q}}}}^{{\mathbf{T}}} {{\varvec{\uplambda}}}_{{{\text{rd}}}}^{{}} = {\mathbf{F}}_{{{\text{rd}}}} } \\ {{{\varvec{\Phi}}}_{r} ({\mathbf{q}},t) = 0} \\ \end{array} } \right. $$

where $${\mathbf{M}}_{{{\text{rd}}}} = \left[ {\begin{array}{*{20}c} {{\mathbf{M}}_{r} } & 0 \\ 0 & {{\mathbf{M}}_{d} } \\ \end{array} } \right]$$, $${\mathbf{C}}_{{{\text{rd}}}} = \left[ {\begin{array}{*{20}c} {{\mathbf{C}}_{r} } & 0 \\ 0 & {{\mathbf{C}}_{d} } \\ \end{array} } \right]$$, $${\mathbf{K}}_{{{\text{rd}}}} = \left[ {\begin{array}{*{20}c} {{\mathbf{K}}_{r} } & 0 \\ 0 & {{\mathbf{K}}_{d} } \\ \end{array} } \right]$$, $${\mathbf{F}}_{{{\text{rd}}}} = \left[ \begin{gathered} {\mathbf{F}}_{r} \hfill \\ {\mathbf{F}}_{d} \hfill \\ \end{gathered} \right]$$, $${{\varvec{\Phi}}}_{{{\text{rd}}{\mathbf{q}}}}^{{\mathbf{T}}} = \left[ {\begin{array}{*{20}c} {{{\varvec{\Phi}}}_{{{\text{r}}q}}^{{\mathbf{T}}} } & 0 \\ 0 & 0 \\ \end{array} } \right]$$, and $${{\varvec{\uplambda}}}_{{{\text{rd}}}}^{{}} = \left\{ \begin{gathered} {{\varvec{\uplambda}}}_{r}^{joint} \hfill \\ 0 \hfill \\ \end{gathered} \right\}$$.

**Figure 6:**
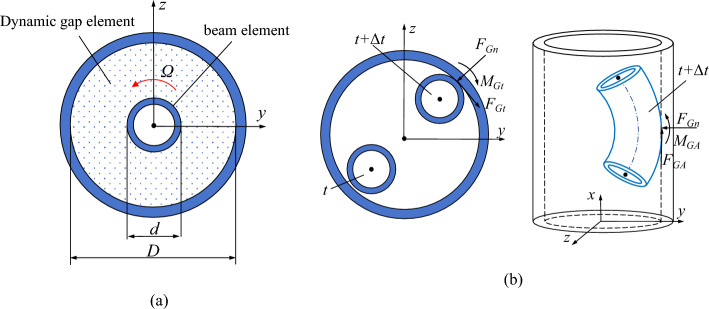
A diagrammatic drawing of **the** dynamic gap element. (**a**) Schematic representation of the dynamic gap element, (**b**) Schematic representation of the contact force during collision.

The interaction between the flexible drill pipe and guide pipe, as well as between the guide pipe and the well bore, is characterized by employing a dynamic gap element. A schematic representation of this dynamic gap element and the corresponding collision contact force can be found in Fig. 6.

The local coordinate system of the gap element should align with both the local coordinate systems of the flexible drill string beam element and guide pipe beam element. The displacement of any node I within the dynamic gap element can be represented as follows:

20 $$ {\mathbf{f}}_{G} {(}t{) = }\left[ {\begin{array}{*{20}c} {v_{i} } & w \\ \end{array}_{i} } \right]^{{\text{T}}} = {\mathbf{N}}_{G} {\mathbf{q}}_{i} $$

where ${\mathbf{q}}_{i}$ represents the displacement array of node I in the flexible drilling tool beam element and ${\mathbf{N}}_{G}$ denotes the shape function matrix of the gap element.

21 $$ {\mathbf{N}}_{G} { = }\left[ {\begin{array}{*{20}c} 0 & 1 & 0 & 0 & 0 & 0 \\ 0 & 0 & 1 & 0 & 0 & 0 \\ \end{array} } \right] $$

The stiffness matrix of the dynamic gap element is determined:

22 $$ {\mathbf{K}}_{G}^{e} { = }{\mathbf{N}}_{G}^{{\text{T}}} G_{k} {\mathbf{N}}_{G}^{{}} $$

where the matrix ${\mathbf{K}}_{G}^{e}$ represents the stiffness of the dynamic gap element and $G_{k}$ denotes its compressive stiffness during contact.

During the operation of the flexible pipe and pipe run, no contact is observed between the pipe run and borehole wall. The dynamic gap elements adhere to kinematic principles, with their velocity determined accordingly:

23 $$ {{\varvec{\upupsilon}}}_{G} { = }\left\{ {\begin{array}{*{20}c} {\upsilon_{T} } \\ {\upsilon_{n} } \\ \end{array} } \right\}{ = }{\dot{\mathbf{f}}}_{G} + \left\{ {\begin{array}{*{20}c} {\dot{\theta }} \\ 0 \\ \end{array} } \right\}\frac{d}{2} $$

The acceleration of the dynamic gap element is:

24 $$ {\mathbf{a}}_{G} { = }\left\{ {\begin{array}{*{20}c} {a_{T} } \\ {a_{n} } \\ \end{array} } \right\}{ = }{\mathbf{\ddot{f}}}_{G} + \left\{ {\begin{array}{*{20}c} {\ddot{\theta }} \\ {\dot{\theta }^{2} } \\ \end{array} } \right\}\frac{d}{2} $$

where $\dot{\theta }$ represents the torsional angular velocity of the flexible drill tool and $\ddot{\theta }$ represents the angular acceleration of the flexible drill tool.

The collision between the flexible drill pipe and the guide pipe, as well as between the guide pipe and the shaft wall, generates reaction forces, resistance distances, and bending moments:

25 $$ \left\{ {\begin{array}{*{20}c} {F_{Gt} { = }\mu_{1} F_{Gn} } \\ {F_{GA} { = }\mu_{2} F_{Gn} } \\ {M_{Gt} { = }\frac{d}{2}F_{Gt} } \\ {M_{GA} { = }\frac{d}{2}F_{GA} } \\ \end{array} } \right. $$

where $F_{Gn}$ is the normal contact force, $F_{Gt}$ is the tangential friction resistance, $F_{GA}$ is the axial friction resistance, $\mu_{1}$ and $\mu_{2}$ are friction coefficients, $M_{Gt}$ is the bending moment caused by the tangential friction resistance, and $M_{GA}$ is the bending moment caused by the axial friction resistance.

The additional forces mentioned above are transformed into the corresponding nodal forces of the gap element:

26 $$ {\mathbf{F}}_{G}^{e} = \left[ {\begin{array}{*{20}c} {F_{GA} } & {F_{Gty} } & {F_{Gtz} } & {M_{Gt} } & {M_{GAy} } & {M_{{GA{\text{z}}}} } \\ \end{array} } \right]^{{\text{T}}} $$

where $F_{Gty}$ and $F_{Gtz}$ are the components of $F_{Gt}$ and $M_{GAy}$ and $M_{{GA{\text{z}}}}$ are the components of $M_{GA}$.

**Figure 7:**
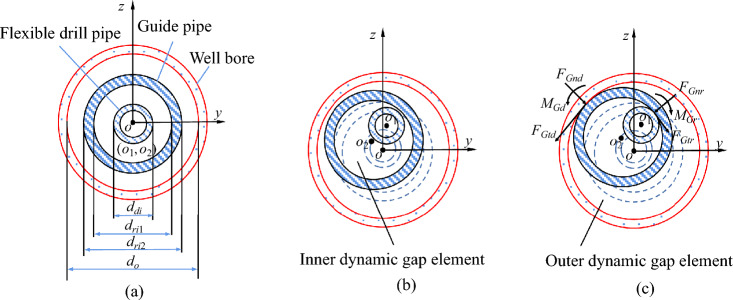
Schematic diagram of the two-layer contact dynamic gap elements. (**a**) initial state, (**b**) free state, (**c**) contact state.

The internal dynamic gap element between the flexible drill pipe and the guide pipe was established, and the external dynamic gap element between the guide pipe and the wall bore was constructed (Fig. 7).

The initial gap of the inner dynamic gap element in any direction is:

27 $$ g_{1} = \frac{1}{2}(d_{ri1} - d_{di} ) $$

The inner dynamic stiffness matrix of the gap elements is:

28 $$ {\mathbf{K}}_{G}^{e} { = }{\mathbf{N}}_{G}^{{\text{T}}} G_{rk} {\mathbf{N}}_{G}^{{}} $$

The nodal force equivalent to the inner dynamic gap element is:

29 $$ {\mathbf{F}}_{rG}^{e} = \left[ {\begin{array}{*{20}c} {F_{rGA} } & {F_{rGty} } & {F_{rGtz} } & {M_{rGt} } & {M_{rGAy} } & {M_{rGAz} } \\ \end{array} } \right]^{{\text{T}}} $$

The equation for the inner power balance of the gap elements is:

30 $$ {\mathbf{K}}_{rG}^{e} {\mathbf{q}}_{r} {(}t{) = }{\mathbf{F}}_{rG}^{e} (t) $$

The gap of the outer dynamic gap element is present in any direction:

31 $$ g_{2} = \frac{1}{2}(d_{o} - d_{ri2} ) $$

The stiffness matrix of the outer dynamic gap element is:

32 $$ {\mathbf{K}}_{Gd}^{e} { = }{\mathbf{N}}_{G}^{{\text{T}}} G_{dk} {\mathbf{N}}_{G}^{{}} $$

The nodal force equivalent to the outer dynamic gap element is:

33 $$ {\mathbf{F}}_{Gd}^{e} = \left[ {\begin{array}{*{20}c} {F_{dGA} } & {F_{dGty} } & {F_{dGtz} } & {M_{dGt} } & {M_{dGAy} } & {M_{dGAz} } \\ \end{array} } \right]^{{\text{T}}} $$

The equilibrium equation governing the outer dynamic gap element is presented:

34 $$ {\mathbf{K}}_{dG}^{e} {\mathbf{q}}_{d} {(}t{) = }{\mathbf{F}}_{dG}^{e} (t) $$

The stiffness matrix of the inner and outer dynamic gap elements is incorporated into the dynamic equation of the flexible drilling tool after coordinate transformation and splicing.

35 $$ \left\{ {\begin{array}{*{20}c} {{\mathbf{M}}_{{{\text{rd}}}} {\ddot{\mathbf{q}}}_{{{\text{rd}}}} (t) + {\mathbf{C}}_{{{\text{rd}}}} {\dot{\mathbf{q}}}_{{{\text{rd}}}} (t) + \left( {{\mathbf{K}}_{{{\text{rd}}}} + {\mathbf{K}}_{{G{\text{r}}}} + {\mathbf{K}}_{{G{\text{d}}}} } \right){\mathbf{q}}_{{{\text{rd}}}} (t) + {{\varvec{\Phi}}}_{{{\text{rd}}{\mathbf{q}}}}^{{\mathbf{T}}} {{\varvec{\uplambda}}}_{{}}^{{}} = {\mathbf{F}}_{{{\text{rd}}}} (t) + {\mathbf{F}}_{{{\text{r}}G}} (t) + {\mathbf{F}}_{{{\text{d}}G}} (t)} \\ {{{\varvec{\Phi}}}_{r} ({\mathbf{q}},t) = 0} \\ \end{array} } \right. $$

The conditions of the contact state are shown in Table 2.

**Table 2:** Contact state conditions.

Free state	Inner-layer contact	Outer-layer contact	Two-layer contact state
$g_{1} > 0$,$g_{2} > 0$	$g_{1} \le 0$,$g_{2} > 0$	$g_{1} > 0$,$g_{2} \le 0$	$g_{1} \le 0$,$g_{2} \le 0$

### Model solving algorithm

Due to the presence of a nonlinear term in the equation governing the dynamics of an articulated flexible drill, both the Newmark method and Newton Raphson method are employed for solving this nonlinear differential equation^21^.

Applying Newmark to Eq. (34) gives the equilibrium equation at the time:

36 $$ {\hat{\mathbf{K}}\mathbf{q}}^{\xi + 1} = {\hat{\mathbf{F}}}^{\xi + 1} $$

where

37 $$ {\hat{\mathbf{K}}} = {\mathbf{K}}_{rd} + {\mathbf{K}}_{Gr} + {\mathbf{K}}_{Gd} + \frac{1}{{\kappa {\Delta }t_{\xi }^{2} }}{\mathbf{M}}_{rd} + \frac{\gamma }{{\kappa {\Delta }t_{\xi }^{{}} }}{\mathbf{C}}_{rd} $$

38 $$ \begin{aligned} {\hat{\mathbf{F}}}^{\xi + 1} = & {\mathbf{F}}_{rd}^{\xi + 1} + {\tilde{\mathbf{F}}}^{\xi + 1} + {\mathbf{M}}_{rd} \left[ {\frac{1}{{\kappa {\Delta }t_{\xi }^{2} }}{\mathbf{q}}_{rd}^{\xi } + \frac{1}{{\kappa {\Delta }t_{\xi } }}{\dot{\mathbf{q}}}_{rd}^{\xi } + \left( {\frac{1}{2\kappa } - 1} \right){\ddot{\mathbf{q}}}_{rd}^{\xi } } \right] \\ & \quad + {\mathbf{C}}_{rd} \left[ {\frac{\gamma }{{\kappa {\Delta }t_{\xi } }}{\mathbf{q}}_{rd}^{\xi } + \left( {\frac{\gamma }{\kappa } - 1} \right){\dot{\mathbf{q}}}_{rd}^{\xi } + \frac{{{\Delta }t_{\xi } }}{2}\left( {\frac{\gamma }{\kappa } - 2} \right){\ddot{\mathbf{q}}}_{rd}^{\xi } } \right] \\ \end{aligned} $$

39 $$ {\tilde{\mathbf{F}}}^{\xi + 1} { = }{\mathbf{F}}_{Gr}^{\xi + 1} + {\mathbf{F}}_{Gd}^{\xi + 1} $$

where ${\Delta }t_{\xi }$ is the time step and $\kappa$ and $\gamma$ are parameters in the Newmark method.

Equation (37) is a nonlinear system of equations that is iteratively calculated by the Newton–Raphson method

40 $$ {\hat{\mathbf{K}}}_{T} {\Delta }{\mathbf{q}}_{rd}^{{\xi {(}\psi {)}}} = {\hat{\mathbf{F}}}_{{}}^{{\xi + 1{(}\psi {)}}} - {\hat{\mathbf{K}}\mathbf{q}}_{rd}^{{\xi + 1{(}\psi {)}}} = {\mathbf{R}}_{rd}^{{\xi + 1{(}\psi {)}}} $$

where

41 $$ {\hat{\mathbf{K}}}_{T} = {\mathbf{K}}_{T} + {\mathbf{K}}_{Gr}^{{\xi + 1{(}\psi {)}}} + {\mathbf{K}}_{Gr}^{{\xi + 1{(}\psi {)}}} + \frac{1}{{\kappa {\Delta }t_{\xi }^{2} }}{\mathbf{M}}_{rd} + \frac{\gamma }{{\kappa {\Delta }t_{\xi } }}{\mathbf{C}}_{rd} $$

42 $$ \begin{aligned} {\hat{\mathbf{F}}}^{{\xi + 1{(}\psi {)}}} = & {\mathbf{F}}^{\xi + 1} + {\tilde{\mathbf{F}}}^{{\xi + 1{(}\psi {)}}} + {\mathbf{M}}_{rd} \left[ {\frac{1}{{\kappa {\Delta }t_{\xi }^{2} }}{\mathbf{q}}_{rd}^{\xi } + \frac{1}{{\kappa {\Delta }t_{\xi } }}{\dot{\mathbf{q}}}_{rd}^{\xi } + \left( {\frac{1}{2\kappa } - 1} \right){\ddot{\mathbf{q}}}_{rd}^{\xi } } \right] \\ & \quad + {\mathbf{C}}_{rd} \left[ {\frac{\gamma }{{\kappa {\Delta }t_{\xi } }}{\mathbf{q}}_{rd}^{\xi } + \left( {\frac{\gamma }{\kappa } - 1} \right){\dot{\mathbf{q}}}_{rd}^{\xi } + \frac{{{\Delta }t_{\xi } }}{2}\left( {\frac{\gamma }{\kappa } - 2} \right){\ddot{\mathbf{q}}}_{rd}^{\xi } } \right] \\ \end{aligned} $$

where $\psi$ is the number of iterations and ${\mathbf{K}}_{T}$ is the tangential stiffness matrix.

The above iterative calculation formula applies the expression for the displacement increment iteration.

43 $$ {\mathbf{q}}_{rd}^{{\xi + 1{(}\psi + 1{)}}} = {\mathbf{q}}_{rd}^{k} + \sum\limits_{j = 0}^{\psi } {\Delta {\mathbf{q}}_{rd}^{{\xi {(}j{)}}} } $$

Moreover,

44 $$ {\mathbf{q}}_{rd}^{{\xi + 1{(0)}}} = {\mathbf{q}}_{rd}^{\xi } $$

### Verification of the numerical method

**Figure 8:**
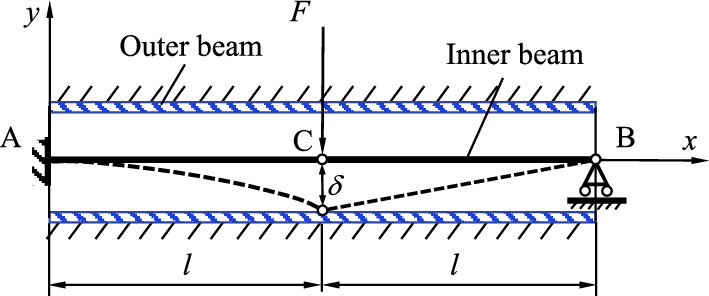
Schematic diagram of the inner and outer beams.

The numerical calculation method in this paper is verified by a numerical example in reference^12^. There is a controllable hinge connection beam inside the beam (Fig. 8). The outer beam is completely fixed. The left end of the inner beam is fully fixed, and the right end is movable hinge support. Point C is subjected to a concentrated load $F(t) = 1000t{ (0} \le t \le 0.08{)}$ (unit N).

The analytical solution of the displacement in the y direction at point C is:

45 $$ \Delta {(}t{) = }\left\{ {\begin{array}{*{20}c} { - \frac{{{7}F{(}t{)}l}}{{{96}EI}}} & {F{(}t{)} \le F_{{\text{r}}} } \\ { - \frac{{{7}F{(}t{)}l}}{{{96}EI}} - \frac{{5}}{{{16}}}\chi l} & {F_{{\text{r}}} \le F{(}t{)} \le F_{{1}} } \\ { - \delta } & {F{(}t{)} > F_{{1}} } \\ \end{array} } \right. $$

Fr is the critical concentration force at the hinge required to reach the rotation limit; F1 is the critical concentration force of contact between the inner beam and the outer beam.

**Figure 9:**
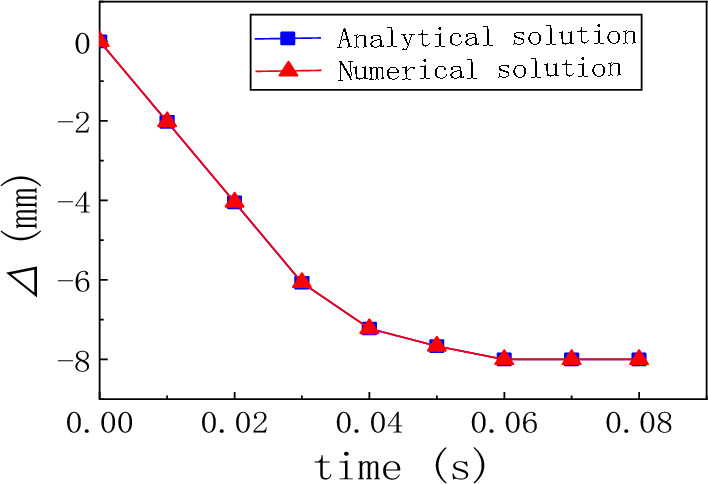
Comparison between the analytical and numerical solutions for the hinge point displacement.

A comparison between the analytical solution and the numerical solution is shown in Fig. 1. As shown in Fig. 9, the maximum error is 0.9%, and the calculation model satisfies the accuracy conditions.

## Results and discussion

The calculation parameters of the numerical simulation are shown in Table 3.

**Table 3:** Dynamic analysis and calculation parameters of flexible drilling tools.

Items	Symbol/unit	Numerical value
Rotate speed	${{\varvec{\Omega}}}$/(r/min)	40
Radius of wellbore curvature	$\rho$/m	3.2
Well inclination angle	$\theta$/°	90
Dynamic friction factor	$\eta$	0.235
Diameter of bit	*D*/m	0.118

### Motion state analysis

**Figure 10:**
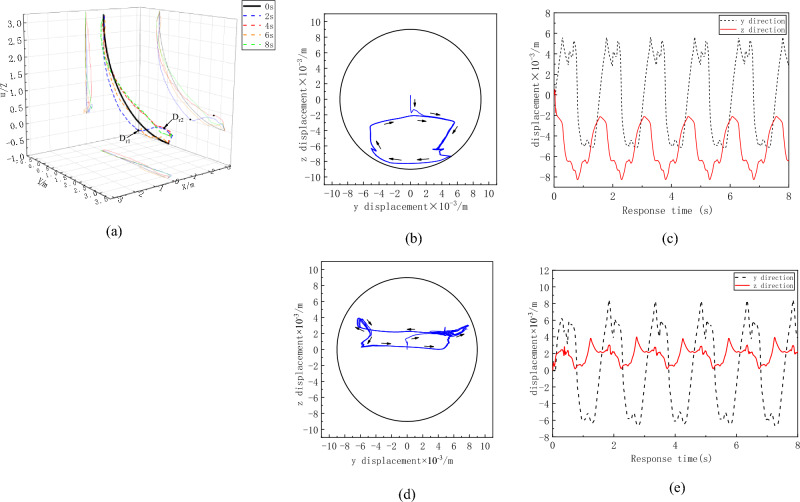
Displacement curve of the flexible drill pipe. (**a**)Overall deformation of flexible drill pipe, (**b**) D_r1_ Motion trajectory, (**c**) D_r1_ Displacement curve with time, (**d**) D_r2_ motion trajectory, (**e**) D_r2_ motion trajectory.

To clearly observe the overall movement trend of the flexible drilling tool, the deformation of the flexible drilling tool is amplified by 50 times. The overall deformation of the flexible drill pipe is shown in Fig. 10. It can be observed from the figures that the deformation of the flexible drill pipe exhibits characteristics of broken line deformation. The flexible drill pipe rotates around the hole, most notably at the D_r1_ and D_r2_ positions. The motion trajectories at the D_r1_ and D_r2_ positions are shown in Fig. 10b and d, respectively. The flexible drill pipe at D_r1_ rotates in the same direction as the applied speed, and the flexible drill pipe at D_r2_ rotates in the opposite direction. In terms of the range of motion, the lateral range of motion in the D_r2_ position is greater. The deformation of the bottom of the flexible drill is more pronounced because the position displacement constraint at the bottom is looser compared to that at the top. This is consistent with the deformation law of flexible drilling tools.

**Figure 11:**
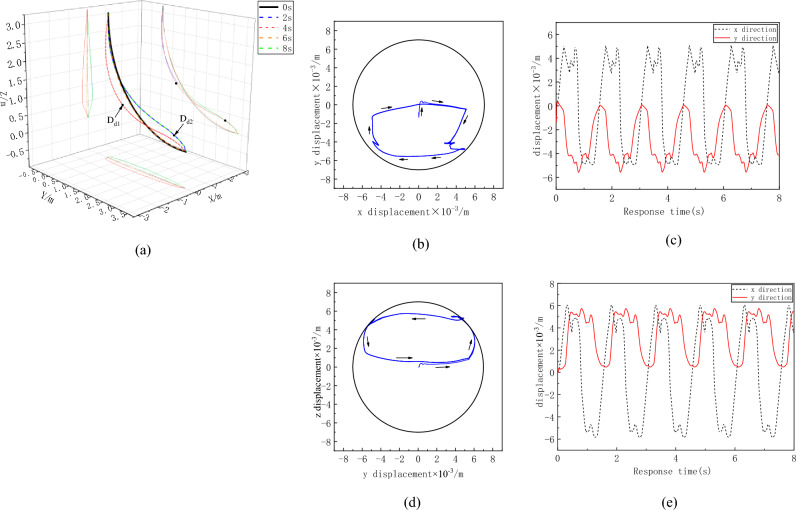
Displacement curve of the guide pipe. (**a**) Overall deformation of guide pipe, (**b**) D_d1_ Motion trajectory, (**c**) D_d1_ Displacement curve with time, (**d**) D_d2_ Motion trajectory, (**e**) D_d2_ Displacement curve with time.

The overall deformation of the guide pipe is shown in Fig. 11a. Compared with that of a flexible drill pipe, the deformation of a guide pipe is smoother. The largest deformations are D_d1_ and D_d2_, and the motion tracks of D_d1_ and D_d2_ are shown in Fig. 11b and d, respectively. Similar to the trajectory of the flexible drill pipe, D_d1_ and D_d2_ move in the same and opposite directions, respectively, of the rotational speed at the top. Although the guide pipe is only subjected to axial load, the whirl phenomenon also appears under the disturbance of the flexible drill pipe.

**Figure 12:**
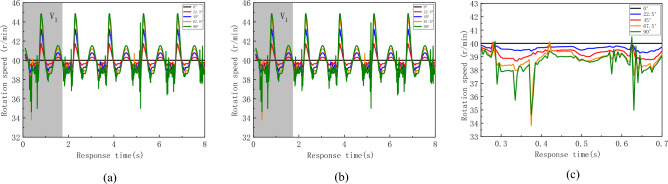
Schematic diagram of the rotation speed. (**a**) The rotate speed of flexible drill pipe changes with time, (**b**) V_1_ section, (c) V_2_ section.

The variation curves of the rotational speed of the flexible drill pipe at well inclination angles of 0°, 22.5°, 45°, 67.5° and 90° with time are shown in Fig. 12. Figure 12b,c show that the rotational speed of the flexible drill pipe is gradually amplified and fluctuates widely with increasing well inclination angle.

**Figure 13:**
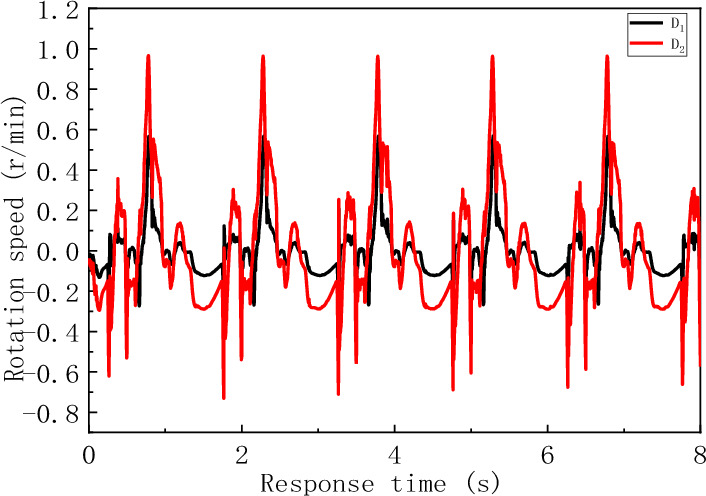
The change in the rotational speed of the guide pipe with time.

The rotational speeds of the guide pipes at positions D_1_ and D_2_ are shown in Fig. 13. Under the influence of the rotation of the flexible drill pipe, the guide pipe has a revolution velocity around the hole, but the revolution velocity is small. The rotation period of the guide tube is the same as that of the flexible drill pipe. Under the influence of the flexible drill pipe and bit weight, the rotational velocity of the guide pipe fluctuates between − 0.74 and 0.97 r/min.

### Collision contact analysis

**Figure 14:**
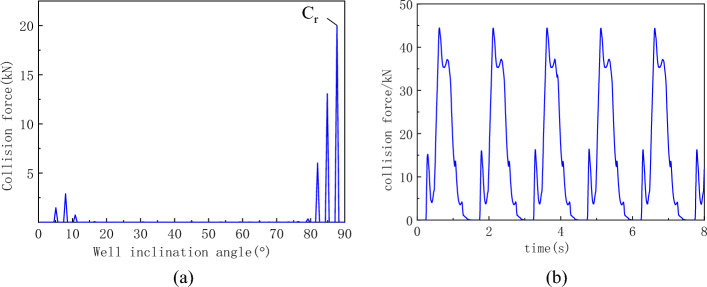
Collision force curves of the flexible drill pipe and guide pipe. (**a**) Effective values of collision forces at various well angles, (**b**) Time history curve at point C_r_.

Considering the randomness of the collision force and acceleration caused by the sampling frequency, their effective values will be used as the basis for analysis in the following text. The collision force between the flexible drill pipe and the guide pipe is depicted in Fig. 14. Figure 14a shows that the intense collisions between the flexible drill pipe and guide pipe are concentrated at the bottom of the hole, reaching a maximum value of 19.97 kN at an inclination angle of 88°, corresponding to the lowermost section of the flexible drill pipe. The temporal evolution of this collision force is depicted in Fig. 14b, revealing periodic characteristics in terms of the collision force between these two pipes at this position. The collision period lasts for 1.5 s, with each individual collision lasting for approximately 1.27 s and a maximum recorded collision force measuring up to 44.42 kN. It can be seen that the impact of the flexible drill pipe is mainly concentrated in the position near the drill bit, which is the vulnerable part.

**Figure 15:**
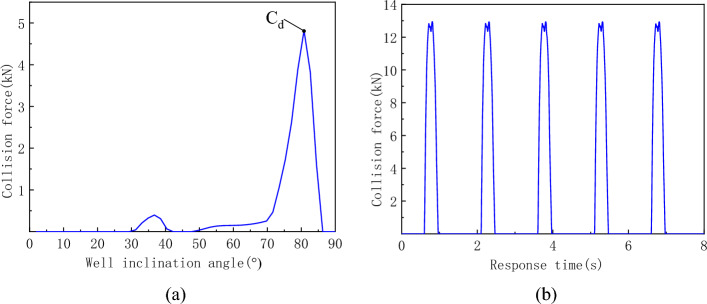
Collision force curve between the guide pipe and shaft wall. (**a**) Effective values of collision forces at various well angles, (**b**) Time history curve at point C_d_.

The collision force curve between the guide pipe and the shaft wall is shown in Fig. 15. Figure 15a shows that severe collisions between the guide pipe and the borehole wall are concentrated at the bottom of the hole, with a maximum value of 4.82 kN and a borehole inclination of 81°. The change curve of the collision force at this position over time is shown in Fig. 15b. Figure 15b shows that the maximum collision force is 12.81 kN, the collision period between the guide pipe and the shaft wall at this position is the same as that between the flexible drill pipe and the guide pipe at the same position, which is 1.5 s, and the duration of each collision is 0.56 s.

**Figure 16:**
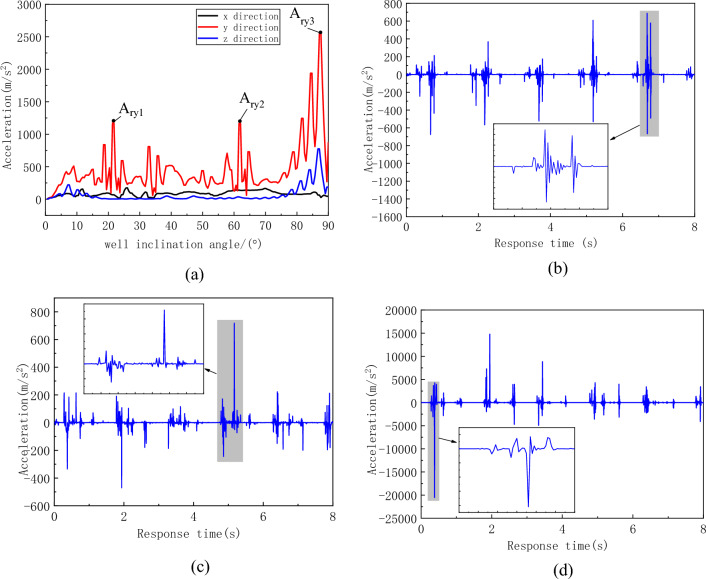
Acceleration curve of the flexible drill pipe. (**a**) Effective values of acceleration at various well angles, (**b**) Time history curve at point A_ry1_, (**c**) Time history curve at point A_ry2_, (**c**) Time history curve at point A_ry3_.

The variation curves of the effective acceleration in the three directions of the flexible drill pipe with respect to the well inclination angle are shown in Fig. 16. The vibration fluctuation in the y direction (transverse direction) of the flexible drill pipe is large, taking the positions A_ry1_, A_ry2_ and A_ry3_ of the acceleration curve in the y direction, as shown in Fig. 16b–d, respectively. The figure shows that the vibration of the flexible drill pipe has a certain periodicity, and the vibration of the flexible drill pipe is close to the collision period. Combined with the collision force curve, it can be concluded that the violent vibration of the flexible drill pipe is caused by the collision.

**Figure 17:**
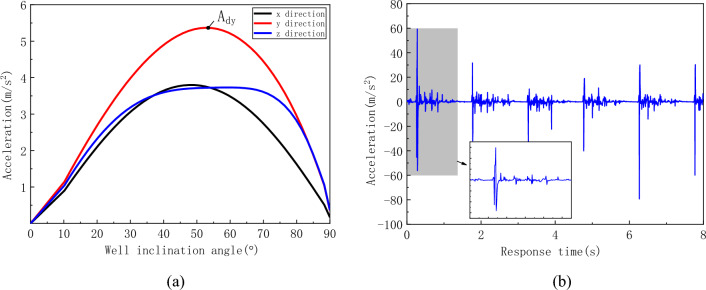
Acceleration curve of the flexible drill pipe. (**a**) Effective values of acceleration at various well angles, (**b**) Time history curve at point A_dy_.

The acceleration curve of the guide pipe is shown in Fig. 17. Although the guide pipe is subjected to greater impact, its vibration is smaller than that of the flexible drill pipe. As can be seen from Fig. 17b, the vibration of the guide pipe is relatively gentle. The reason for this difference is that the guide pipe is a continuous body in structure, while the flexible drill pipe is a multi-stage hinged multi-body system, so the vibration of the flexible drill pipe is more intense.

## Conclusions

1. The numerical results are compared with the theoretical results to verify the accuracy of the numerical method. The error between the calculated displacement results and theoretical results is 0.9%.
2. In the wellbore, flexible drilling tools have a whirl phenomenon, which may increase friction during drilling. It is suggested to place a centralizer on the outer wall of the flexible drilling tool to reduce the dynamic friction during drilling. Under the interaction between the guide tube and the flexible drill pipe, a whirl phenomenon occurs in the flexible drill pipe, resulting in increased dynamic friction during drilling and leading to greater wear. Although the guide tube in the inclined section only experiences axial force, the vortex phenomenon introduces a more complex stress state that necessitates consideration of possible torsional forces in its analysis and design.
3. The severe vibration of flexible drilling tools is mainly caused by flexible drilling pipe. Throughout the drilling procedure, horizontal transverse vibrations are predominantly observed in flexible drilling tools. Conversely, flexible drill pipes display heightened levels of vibration when compared to guide pipes, whereas guide pipes showcase a more consistent vibration pattern.
4. The connection point between the flexible drill pipe and guide tube experiences substantial and regular collision forces, suggesting comparable motion states for both components. The most intense collisions are observed at two locations: where the flexible drill pipe meets the guide pipe, and where the guide tube interacts with the shaft wall near the lower end of a flexible drill pipe joint. These recurring impacts pose a risk of joint failure.

## Data Availability

The data that support the findings of this study are available from [Downhole Service Company of Daqing Oilfield Limited Company] but restrictions apply to the availability of these data, which were used under license for the current study, and so are not publicly available. Data are however available from the authors upon reasonable request and with permission of [Downhole Service Company of Daqing Oilfield Limited Company]. Author: Zhiqiang Lin Email: 919960766@qq.com.
